# The Brons-Mulié analysis as a decision-making tool for preoperative surgical simulation in orthognatic surgery

**DOI:** 10.3389/froh.2025.1511342

**Published:** 2025-02-19

**Authors:** Sebastian Böttger, Yannick Nowak, Anita Cornelia Windhorst, Katharina Klaus, Sabine Ruf, Christina Bäcker, Eva May Schraml, Nina Danevitch, Rob Mulié, Hans-Peter Howaldt, Sameh Attia

**Affiliations:** ^1^ Department of Oral and Maxillofacial Surgery, Justus-Liebig-University Giessen, University Hospital Giessen, Giessen, Germany; ^2^ Institute of Medical Informatics, Justus-Liebig-University Giessen, Giessen, Germany; ^3^ Department of Orthodontics, Justus-Liebig-University Giessen, Giessen, Germany; ^4^ Orthodontist, Orfeokliniek, Zoetermeer, Holland; ^5^ Department of Periodontology, Oral Medicine and Oral Surgery, Charité—Universitätsmedizin Berlin, Berlin, Germany

**Keywords:** Brons-Mulié analysis, orthognathic surgery, facial harmony, cephalometry, profile photography

## Abstract

**Objectives:**

Sufficient preoperative planning represents an essential component for the success of orthognathic surgery. Using various analysis methods, dysmorphic areas can be reliably identified and addressed during the planning procedure.

**Methods:**

Brons-Mulié analysis was used to examine profile photographs before and after orthognathic surgery. The attainment of normal values was interpreted as the achievement of facial harmony of the various facial proportions. By comparing the pre- and postoperative analysis, a control of the outcome quality of the orthognathic procedures was performed.

**Results:**

In a total of 160 patients aged 13 to 61 years, the preoperative analysis could be compared with the postoperative Brons-Mulié analysis. Postoperative, facial harmony was found for the vertical dimension in 99 cases (62%), for the upper lip dimension in 95 cases (59%), for the lower lip dimension in 138 cases (86%), and for the chin dimension in 118 cases (74%). This corresponded to an improvement of 20% in the vertical dimension, of 27% in the area of the lower lip and of 6% in the area of the chin. The upper lip area showed a slight deterioration of 7%.

**Conclusion:**

Despite preoperative planning of orthognathic surgery with Brons-Mulié analysis, postoperative results show an overall improvement but not perfection. Even by applying the method, it remains a challenge to achieve perfect facial harmony.

## 1 Introduction

Beauty and esthetics play an essential role in planning and implementation of orthognathic surgery. The objective is not only to transfer the bite into a neutral and stable occlusion, but also to create an overall esthetic and appealing appearance of the patient (1, 2). But how is esthetics defined and how can it be measured? How and to what extent should the dental arches be displaced in order to achieve not only a proper occlusal result but also an equally favorable esthetic appearance of the patient's face? In the frontal view of the face, parallelism of the bi-pupillary line and the occlusal plane as well as symmetry of the facial halves certainly play an essential role (3–5). Canut et al. describe that the “beauty of the human face”, however, depends primarily on a balance of the three prominent facial features nose, lips and chin (6). Reuther even refers to these features as the “facial esthetic triad” and thus gives them a decisive importance in facial analysis prior to orthognathic surgery (7). Brons-Mulié's soft tissue analysis, which can be performed on both cephalometric radiographs and profile photographs of the head, precisely analyzes these structures in the vertical and sagittal dimensions of the face (8). Facial harmony is achieved if the proportions of the anatomical structures to each other approximately correspond to the golden ratio (8). Since the nose as well as the lips and the chin can be strongly influenced by orthognathic surgery, the aim of the surgical procedure should be to bring these facial components into a condition of facial harmony as far as possible (8). Although in the end esthetics is always the result of a subjective assessment, achieving the proportions of facial harmony ultimately leads to a comprehensible, objective esthetics, based on the reproducible mathematical procedure of the Brons-Mulié analysis (8–10). Thus, it can be applied as an auxiliary tool to plan a harmonious facial profile in orthognathic surgery, as described by Freihofer et al. and Mooren (9, 10). Gu et al. were able to show that the lateral attractiveness of the face correlates well with frontal attractiveness. Thus, in case of a harmonious facial profile also good frontal esthetics can be expected (11). In addition, Brons-Mulié analysis can also be applied postoperatively to assess and to improve the quality of surgical results.

The question arises how often facial harmony can be achieved or at least be improved in the context of orthognathic surgery. Furthermore, the question arises if the Brons-Mulié analysis is suitable as a decision-making tool in preoperative surgical simulation.

## 2 Materials and methods

### 2.1 Planning procedure for orthognathic surgery

Preoperative simulation of surgery has been a standard procedure in orthognatic surgery for many years (12). This simulation can be performed either analogously using an articulator or digitally by computer programs (13). In the present study, all surgical simulations were performed using the 3D-OSS articulator according to Krenkel and Lixl (14). This device allows translational as well as rotational movements in all three dimensions on the basis of single-articulated plaster models. Thus, in particular esthetic aspects can be taken into account. Mock-up surgery was performed considering facial photographs, cephalometric examinations, and Brons-Mulié analysis of profile photographs of the face.

### 2.2 Evaluation of profile photographs

Profile photographs were taken with a digital SLR camera in front of a blue background with a distance of about 150 cm. The digital images were printed out on an A4 page with a color printer in order to be able to carry out the soft tissue analysis according to Rob Mulié and Rijnko Brons using a pencil and a geo-triangle. In this way, the face is imaged approximately on a scale of 1:1 and can therefore also be compared well with the cephalometric images.

### 2.3 Brons-Mulié analysis

**Figure 1:**
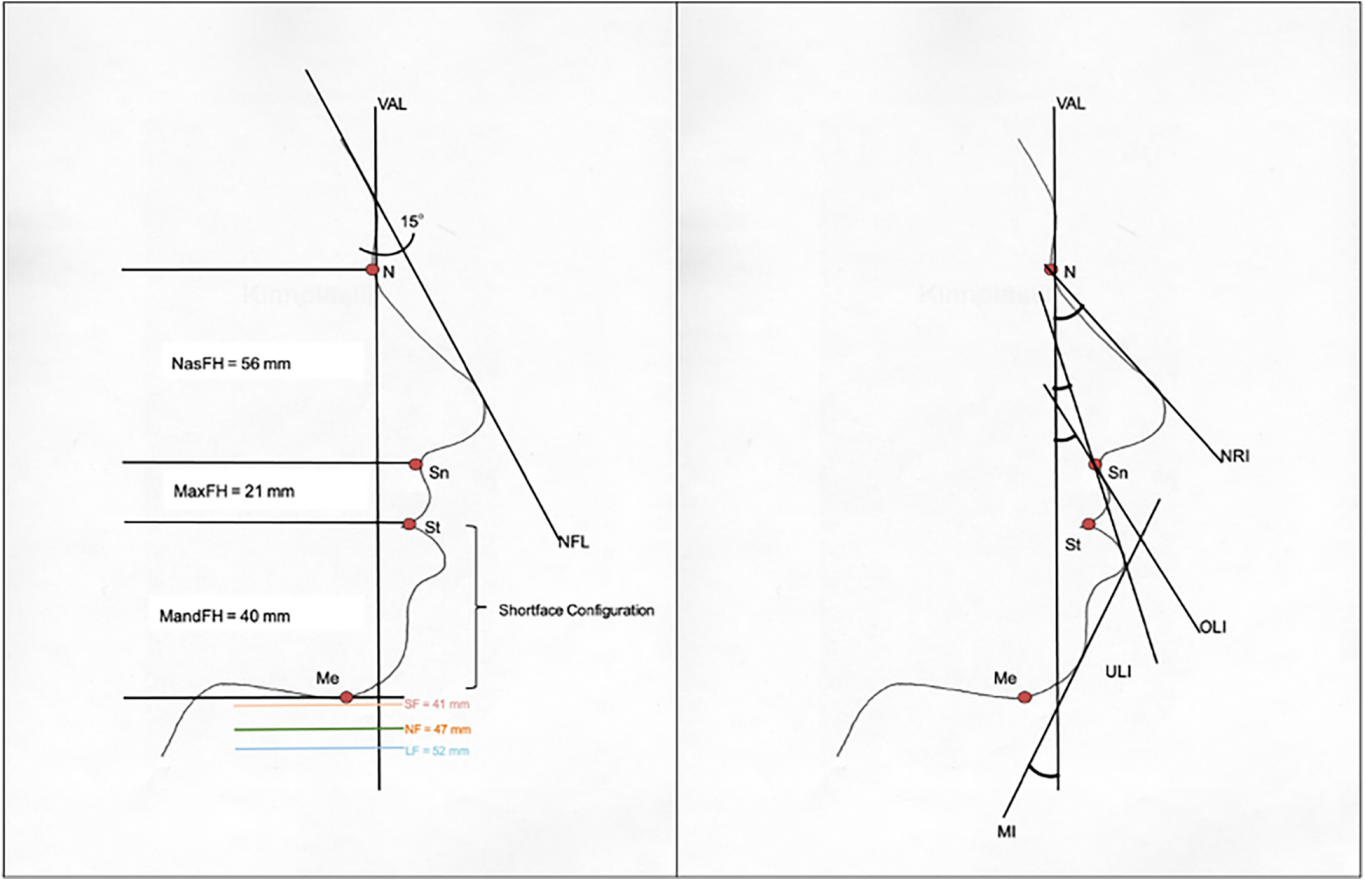
Brons-Mulié analysis in the vertical (left) and sagittal dimension (right). NFL, nasofrontal line; VAL, vertical analysis line; NasFH, nasofacial height; MaxFH, maxillofacial height; MandFH, mandibulofacial height; SF, shortface configuration limit; LF, longface configuration limit; NF, normalface—optimal value for MandFH; NRI, nasal bridge inclination; OLI, upper lip inclination; ULI, lower lip inclination; MI, mandibula inclination; N, nasion; Sn, subnasale; St, stomion; Me, menton.

**Figure 2:**
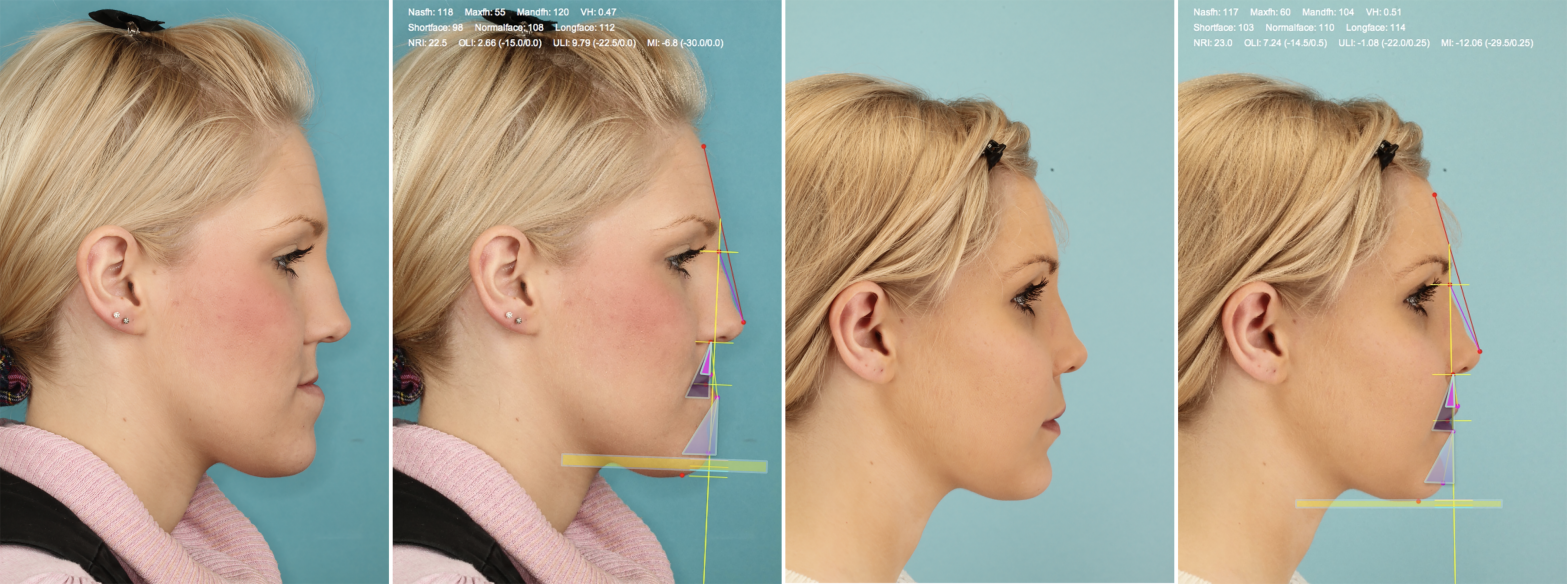
Brons-Mulié's analysis of a patient with class III malocclusion pre- and postoperatively. The yellow area shows the most suitable range for the vertical height. The triangles show the optimal areas for the inclination of the upper lip, the lower lip and the mandible.

Brons-Mulié soft tissue analysis was performed for mock-up surgery approximately six weeks prior to surgery by the surgical team. For quality assurance, soft tissue analyses were repeated by the authors, using postoperative profile photographs, which were taken approximately six months after the operative intervention. As described by Brons (8), the nasal frontal line was drawn for this purpose and a vertical analysis line was drawn at an angle of 15 degrees to the caudal direction (Figure 1). Concerning the vertical dimension, the nasofacial height (length of the nose between the nasion and the subnasal point) and the maxillofacial height (length of the upper lip) were determined. According to Rob Mulié and Rijnko Brons, an optimal value for the mandibulofacial height and a normal range with an upper and lower threshold were calculated from the ratio of these heights. In this context, the lower face was classified as a short face configuration if it was too short and as a long face configuration if it was too long. In the sagittal dimension, the angles between the vertical analysis line and the orientation of the upper lip (upper lip inclination: OLI), the lower lip (lower lip inclination: ULI) and the soft tissue pogonion (Mandibula inclination: MI) were determined. The normal range of these values with an upper and a lower threshold was determined depending on the nasal bridge inclination according to Rob Mulié and Rijnko Brons (8). A too retrogenous profile was described as a dorsal characteristic, a too progenic profile as a ventral characteristic. Facial harmony could be considered if the parameters of the vertical and sagittal dimensions were within the normal range. Figure 2 shows the Brons-Mulie analysis of a patient with a Class III malocclusion pre- and postoperatively.

### 2.4 Statistical methods

Statistical analysis of this retrospective, expoloratory and descriptive study was performed using “Microsoft Excel” and the program “Statistical Package for the Social Sciences” (SPSS®) for Microsoft Windows in collaboration with the Research Group for Medical Statistics of the Justus Liebig University Giessen. All patients who underwent orthognathic surgery between February 2002 and February 2014 and for whom complete documentation was available at follow-up were included in the study. The preoperative and postoperative Brons-Mulié analyses of all patients were compared. Fisher's exact test was used to check whether the vertical (MandFH) and sagittal (OLI, ULI, MI) analysis values could be transferred to or kept within the normal range (=success) or not (=failure). In addition, logistic regression was performed to determine which parameters had the strongest influence on success or failure. All tests were performed with a significance level of *p* < 0.05. Power analysis was carried out with an alpha of 5%, and an odds ratio of 2. In this way, a power of 0.62 to 0.86 can be achieved in dependence on the preoperative level of facial harmony (Supplementary Table S3).

## 3 Results

A total of 160 patients treated with orthognathic surgery between February 2002 and February 2014 were analyzed in this retrospective study. 99 patients (62%) were female and 61 patients (38%) were male. Age at the time of surgery ranged from 13 to 61 years with a median of 23 years. 64 patients (40%) were treated for Class II malocclusion and 92 patients (57.5%) for Class III malocclusion. The remaining patients underwent surgery due to a frontal open bite in Class I occlusion. 123 of 160 patients (76.9%) underwent bimaxillary surgery, 24 patients (15%) underwent bi-sagittal split osteotomy of the mandible, and 13 patients underwent Le Fort 1 osteotomy (8.1%).

### 3.1 Achievement of facial harmony

**Figure 3:**
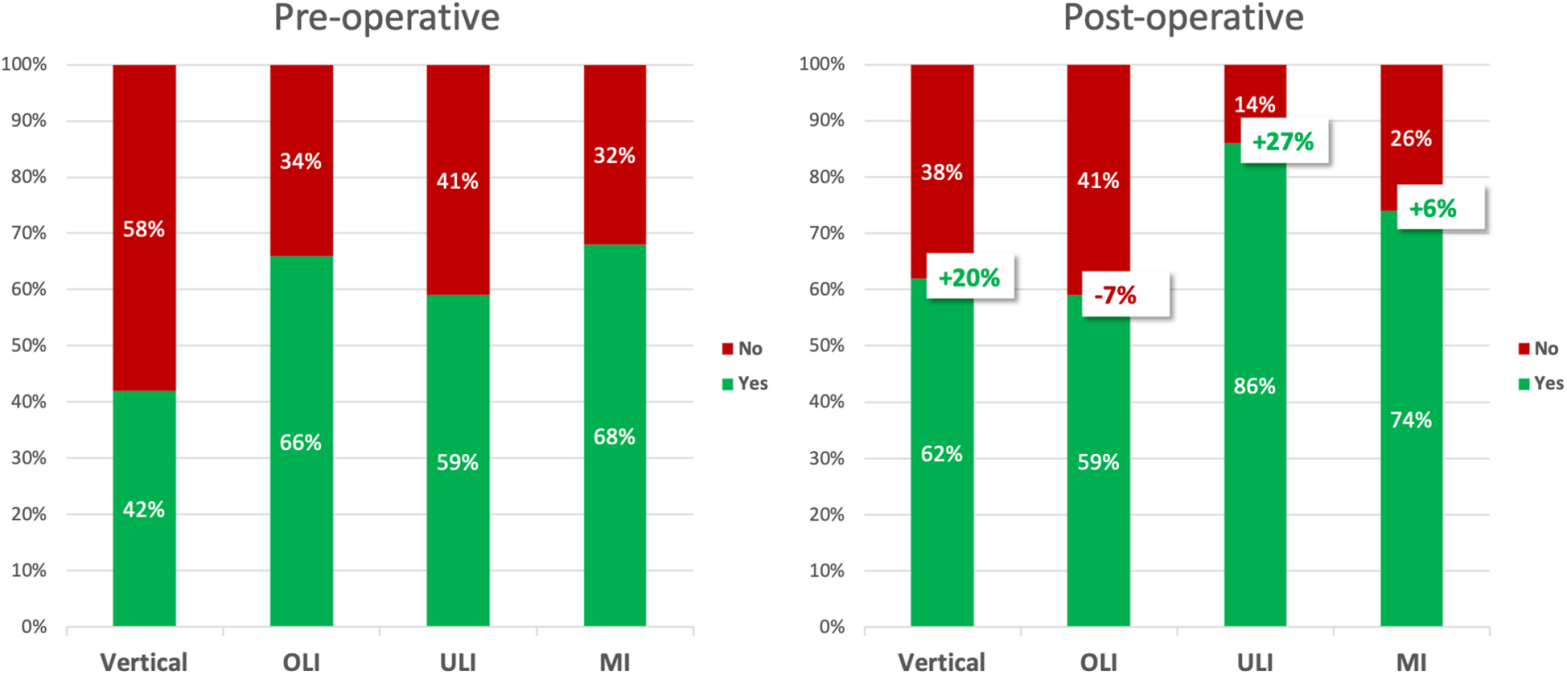
Improvement of facial harmony before and after orthognathic surgery. Vertical, vertical dimension; OLI, upper lip inclination; ULI, lower lip inclination; MI, mandibula inclination.

The examination for the presence of facial harmony was performed in the vertical and sagittal dimensions (OLI, ULI, MI). As expected, facial harmony was seen preoperatively in part of the cases only. Postoperatively, an overall improvement of the facial profiles was observed. However, isolated observation of the upper lip inclination (OLI) even showed a slight deterioration. Table 1 and Figure 3 show the success rates in terms of achieving facial harmony as a result of orthognathic surgery.

**Table 1:** Prevalence of facial harmony before and after surgery in 160 patients.

Facial Harmony	Pre-operative			Post-operative		
	Yes	No		Yes	No	
Vertical	67	93	42%	99	61	62%
OLI	106	54	66%	95	65	59%
ULI	95	65	59%	138	22	86%
MI	109	51	68%	118	42	74%

Facial harmony	Improvement	Aggravation	Odds ratio	CI (lower)	CI (upper)	*p*
Vertical	44	12	5.05	2.31	11.77	<0.01
OLI	21	32	3.60	1.73	7.66	<0.01
ULI	47	4	8.59	2.63	36.90	<0.01
MI	23	14	8.12	3.51	19.64	<0.01

### 3.2 Factors influencing success

Figure 3 shows that although orthognathic surgery was able to enhance facial harmony, the overall success rate was only moderately improved. Therefore, the question arises which patients or constellations may not have been adequately corrected. Table 2 shows the distribution of success and failure depending on pre-operative classification according to Angle. In this context, the Fisher's exact test showed a significant result for the vertical dimension and the mandibula inclination (Table 2). Thus, post-operatively, almost half of the patients with class III showed missing facial harmony in the vertical dimension and almost half of the patients with class II showed missing facial harmony with respect to the Mandibula inclination.

**Table 2:** Facial harmony depending on angle class.

	Angle class	Yes	(%)	No	(%)	Total	p*
Vertical	Class I	3	75.0%	1	25.0%	4	
	Class II	47	73.4%	17	26.6%	64	
	Class III	49	53.3%	43	46.7%	92	
	Total	99	61.9%	61	38.1%	160	*p* = 0.025*
OLI	Class I	3	75.0%	1	25.0%	4	
	Class II	40	62.5%	24	37.5%	64	
	Class III	52	56.5%	40	43.5%	92	
	Total	95	59.4%	65	40.6%	160	*p* = 0.623
ULI	Class I	4	100.0%	0	0.0%	4	
	Class II	57	89.1%	7	10.9%	64	
	Class III	77	83.7%	15	16.3%	92	
	Total	138	86.2%	22	13.8%	160	*p* = 0.716
MI	Class I	4	100.0%	0	0.0%	4	
	Class II	34	53.1%	30	46.9%	64	
	Class III	80	86.9%	12	13.1%	92	
	Total	118	73.8%	42	26.2%	160	*p* < 0.001*

Fisher's exact test showed a significant result for the vertical dimension and the Mandibula inclination (*).

In order to investigate which factors mostly influence success or failure, a logistic regression analysis was performed in which the variables Angle Class, vertical height, upper lip inclination, lower lip inclination, Mandibula inclination, gender, surgery type (Le Fort 1/BSSO/Bimax) and age were included. The best model to explain success was selected using the Akaike criterion (Table 3).

**Table 3:** Logistic regression analysis using the variables angle class, vertical height (vertical), upper lip inclincation (OLI), lower lip inclination (ULI), mandibula inclination (MI), gender, surgery type (le fort 1/BSSO/Bimax.) and age. Akaike criterion was used to select the variables for the best model to explain postoperative success in the four dimensions (vertical dimension, upper lip inclination, lower lip inclination and mandibula inclination).

Success	Df	Deviance	Resid. Df	Resid. Dev	Pr(>Chi)	
Vertical Dimension						
Pre-op. Vertical	2	24.9	157	187.79	<0.001	***
Pre-op. OLI	2	6.53	155	181.27	0.038	*
Pre-op. MI	2	7.26	153	174.01	0.027	*
Mandibula-Inclination						
Pre-op. MI	2	31.00	157	153.21	<0.001	***
Gender	1	1.86	156	151.36	0.173	
Surgery type	2	9.23	154	142.13	0.01	**
Upper Lip-Inclination						
Pre-op. OLI	2	14.19	157	201.96	0.001	***
Pre-op. MI	2	6.94	155	195.02	0.031	*
Pre-op. ULI	2	11.85	153	183.17	0.003	**
Lower Lip-Inclination						
Pre-op. ULI	2	18.56	157	109.57	<0.001	***
Gender	1	3.29	156	106.28	0.07	.
Age	1	3.43	155	102.85	0.064	.

In particular, the preoperative mandibulofacial height was shown to determine success in the vertical direction. Thus, a preoperative long face constellation was associated with a high postoperative failure rate in the vertical dimension (Tables 3, Table 4). Preoperative decreased and increased Mandibula inclination was shown to be most predictive for success of the Mandibula inclination. Table 4 shows that only few of the patients with these constellations show facial harmony of the Mandibula inclination postoperatively. With regard to the lower lip inclination, increased or decreased inclination of the lower lip itself were the main factors determining success.

**Table 4:** In addition to Table 3, the logistic regression analysis examined the individual characteristics of the variables with regard to their influence on success.

Success vertical	Estimate	Odds Ratio	CI (lower)	CI (upper)	Std. Error	z value	Pr(>|z|)
(Intercept)	2.06	7.82	3.76	18.82	0.41	4.97	<0.001
Pre-op. vertical: SF	−0.59	0.56	0.18	1.71	0.56	−1.04	0.298
Pre-op. vertical: LF	−2.17	0.11	0.05	0.27	0.45	−4.79	<0.001
Pre-op. OLI: H	−1.01	0.36	0.14	0.9	0.47	−2.15	0.031
Pre-op. OLI: L	0.98	2.67	0.81	10.73	0.65	1.51	0.13
Pre-op. MI: H	−0.06	0.94	0.26	3.42	0.64	−0.09	0.929
Pre-op. MI: L	−1.33	0.26	0.09	0.7	0.51	−2.61	0.009
Success MI	Estimate	Odds Ratio	CI (lower)	CI (upper)	Sth. Error	z value	Pr(>|z|)
(Intercept)	18.47		0.00		1048.17	0.02	0.986
Pre-op. MI: H	−1.75	0.17	0.05	0.60	0.63	−2.77	0.006
Pre-op. MI: L	−2.19	0.11	0.04	0.28	0.49	−4.47	<0,001
Gender: female	−0.78	0.46	0.18	1.13	0.47	−1.67	0.095
BSSO	−15.25	0.00	0.00		1048.17	−0.01	0.988
Bimax.	−16.34	0.00			1048.17	−0.02	0.988
Success OLI	Estimate	Odds Ratio	CI (lower)	CI (upper)	Sth. Error	z value	Pr(>|z|)
(Intercept)	0.95	2.57	1.53	4.49	0.27	3.46	0.001
Pre-op. OLI: H	−1.09	0.34	0.12	0.88	0.5	−2.19	0.029
Pre-op. OLI: L	−1.15	0.32	0.10	0.95	0.57	−2.03	0.042
Pre-op. MI: H	2.08	7.98	2.02	42.03	0.76	2.75	0.006
Pre-op. MI: L	0.70	2.01	0.80	5.44	0.48	1.44	0.15
Pre-op. ULI: H	−0.96	0.38	0.16	0.93	0.45	−2.12	0.034
Pre-op. ULI: L	−1.98	0.14	0.03	0.5	0.69	−2.87	0.004
Success ULI	Estimate	Odds Ratio	CI (lower)	CI (upper)	Sth. Error	z value	Pr(>|z|)
(Intercept)	1.27	3.56	0.53	23.21	0.95	1.34	0.179
Pre-op. ULI: H	−2.24	0.11	0.03	0.34	0.63	−3.54	<0,001
Pre-op. ULI: L	−2.74	0.06	0.01	0.3	0.8	−3.42	0.001
Gender: female	0.98	2.67	0.99	7.59	0.51	1.91	0.056
Age	0.06	1.06	1.00	1.15	0.04	1.69	0.092

SF, short face; LF, Long face; H, too high; L, too low; BSSO, bisagittal split osteotomy; Bimax, bimaxillary sugery.

## 4 Discussion

A careful planning procedure is essential to ensure favorable surgical outcome in orthognatic surgery (12). It should not only lead to a stable occlusion, but also to a functionally and esthetically acceptable overall result (2, 9, 10, 12, 15). Steenen reported that correction of a disharmonious face is at least as important to patients as oral function recovery (16). Brucoli et al. pointed out that the creation of harmonious facial aesthetics can have a positive effect on patients' compliance and psychological status (17), and Wang et al. also reported positive effects of orthognathic surgery on patients' psychological well-being (18). This further emphasizes the enormous importance of facial esthetics in orthognathic surgery.

The esthetic appearance of the face depends on symmetry, averageness and the proportions of different parts of the face to each other (5, 9). Furthermore, it is evident that lateral attractiveness of the face correlates well with frontal attractiveness (11). Since orthognathic surgery can have a significant impact on facial profile in both, the vertical and sagittal dimension of the face, Freihofer and Mooren recommended defining an aimed-at profile line prior to orthognathic surgery, to which the postoperative result on the patient should approximate as closely as possible (9). But they were also able to show that free artistic drawing of such an aimed-at profile leads to strong variations even among experts and that the result therefore depends highly on individual preferences (9). Thus, Freihofer and Mooren recommend the use of the Brons-Mulié analysis to determine an aimed-at profile as objectively as possible in order to achieve reproducible and attractive postoperative soft tissue esthetics (9, 10).

With the concept of facial harmony, the Brons-Mulie analysis offers a simple tool for assessing facial esthetics, which can be used in addition to standard cephalometric analysis in the planning procedure prior to orthognathic surgery (8). In the vertical dimension the analysis offers a valuable supplement which allows a direct assessment of the vertical facial height. In the sagittal dimension the tool provides an evaluation of the soft tissue pogonion which in contrast to cephalometric analysis is independent of the angulation of the anterior cranial base (8). Further, the tool complements standard cephalometric analysis with an assessment of the lip position.

In the present study, a preoperative Brons-Mulié analysis was carried out preoperatively for all 160 patients in order to achieve an approximately harmonious profile line by performing orthognathic surgery. The outcome was assessed with a second postoperative Brons-Mulié analysis of the face. Data of this work show that despite preoperative application of the Brons-Mulie analysis as a decision-making tool, postoperative facial harmony in the vertical and sagittal dimension is not easy to achieve in every case. Table 1 and Figure 1 show that only the vertical dimension and the lower lip inclination could be markedly improved by 20% and 27%, respectively. In contrast, the Mandibula inclination could only be improved by 6% and the upper lip inclination even showed a slight deterioration of 7%. This could be due to the fact that the Brons-Mulié analysis can indeed visualize preoperative problems, but cannot determine exactly how many millimeters or degrees must be corrected in order to achieve the normal range of values. Furthermore, it is usually not possible to change a single parameter without influencing other parameters. For example, an elongated chin cannot be moved arbitrarily far cranially at the expense of the gingival smile line. Thus, sometimes values outside the normal range have to be accepted during planning and surgery.

Nevertheless, by objectifying esthetic problems and limitations for surgical movements, the Brons-Mulié method can help to favorably influence planning and surgical outcome. For example, in case of a low Mandibula inclination in class II patients, there is clear evidence that surgical advancement of the chin results in a better esthetic outcome (19–22). In the case of a severe chin retraction, the Brons-Mulié analysis can help to change a surgical plan from a pure mandibular advancement to a bimaxillary procedure. Using bimaxillary surgery, the chin can be moved further anteriorly, also by rotating the bimaxillary block around a transversal axis (23). Thus, an esthetically more favorable result can be achieved in many cases (24). Another example is the long face constellation with an increased mandibular inclination, which can be typically seen in Class III patients (25). In such cases, Brons-Mulié analysis may indicate the need to perform a bimaxillary procedure with a rotation of the bimaxillary block and a moderate setback of the mandible instead of a pure advancement of the maxilla, which even leads to an esthetically more favorable result of the facial profile (26, 27).

But the Brons-Mulié analysis is not only suitable as a decision-making tool. Using Fisher's exact test (Table 2) and logistic regression analysis (Tables 3, 4 and Figure 3) it was possible to show that certain initial conditions were obviously more difficult to correct by orthognathic surgery than others.

**Figure 4:**
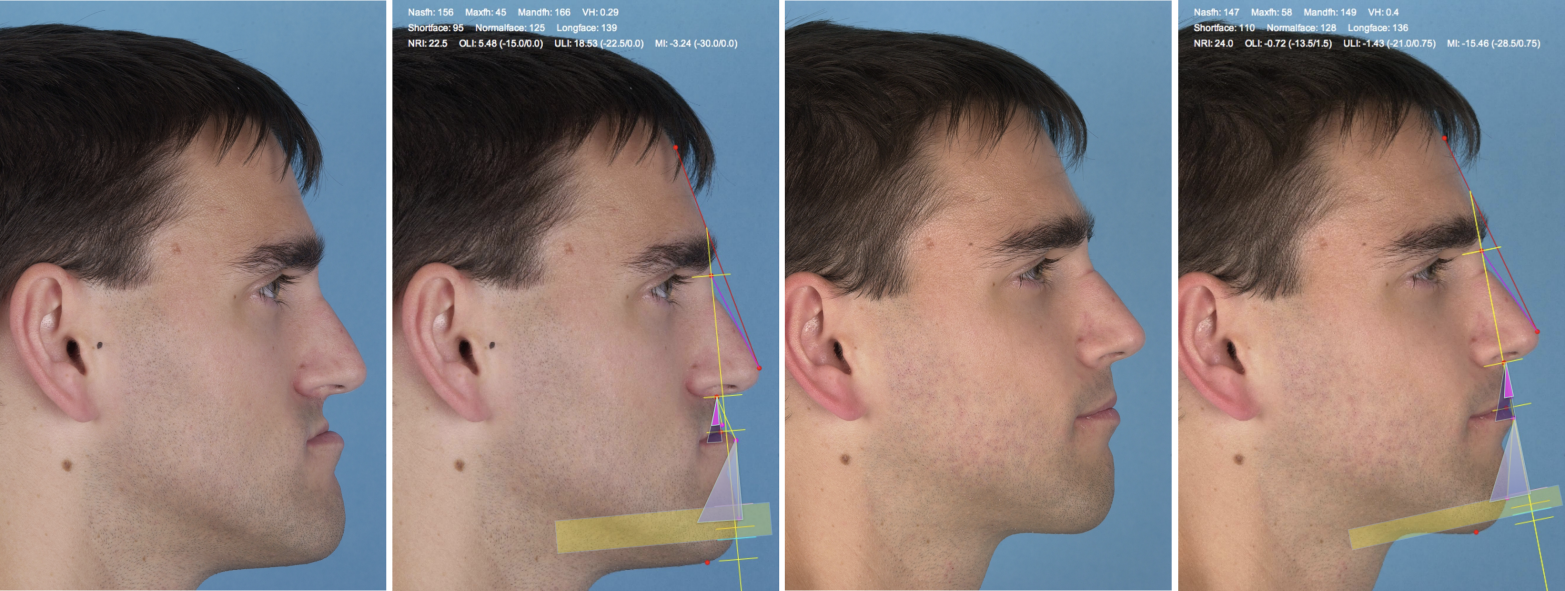
Brons-Mulié analysis of a patient with class III malocclusion pre- und postoperatively. The mandibula inclination could be optimized into the normal range. Despite the significant improvement of the facial profile, the long face constellation in the vertical dimension persisted.

**Figure 5:**
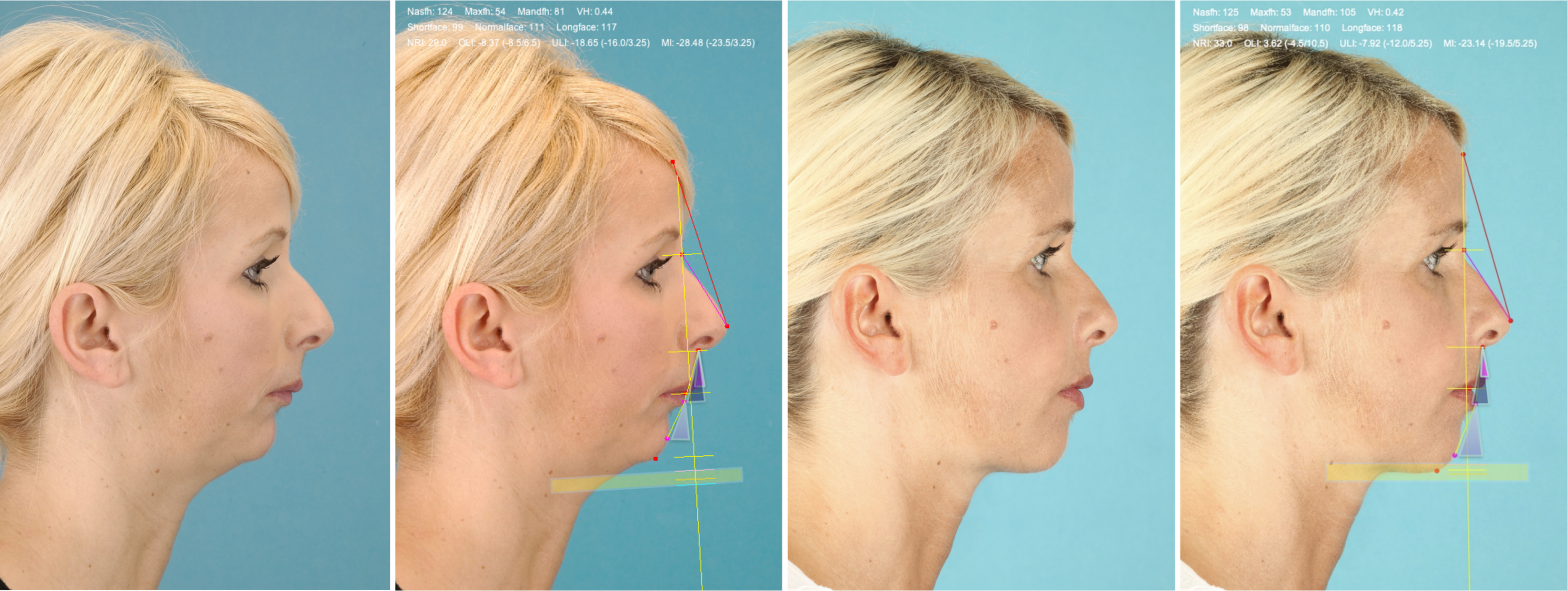
Brons-Mulié analysis of a patient with class II malocclusion pre- and postoperatively. The vertical height could be optimized into the normal range. Despite the significant improvement of the facial profile, the mandibula inclination could not be corrected into the normal range.

Table 3 shows that patients with an Angle Class III could often not be corrected sufficiently in the vertical dimension, while Class II patients often could not be treated successfully in the sagittal dimension with respect to the Mandibula inclination. Looking at the results of the logistic regression analysis, it becomes apparent that a preoperative long face constellation (Table 4: Success vertical/Pre-op. vertical: LF) and a preoperatively reduced Mandibula inclination (Table 4: Success MI/Pre-op. MI: L) had a significant influence on success. Odds ratio for both preoperative conditions was only 0.11, meaning that these constellations could only be corrected into the normal range with a significantly reduced probability. Thus, one constellation that was difficult to correct was the typical class III patient with a characteristic long face situation due to his progeny. Since bimaxillary surgery is often performed in anterior and caudal direction to protect the upper airway (28) and at the same time impaction of the jaws against the cranial base is complex and rather rarely performed, it becomes clear that it is difficult to sufficiently reduce an overly long lower face within the scope of bimaxillary surgery alone (Figure 4). The same applies to a constellation of a too low mandibula inclination. Although the pogonion can be brought much further anteriorly by bimaxillary surgery, in many cases it cannot be brought forward enough to ensure that the Mandibula inclination actually reaches the normal range (Figure 5). Both problems can be solved by an additional genioplasty (29), which can be performed as a bone reducing procedure in case of long face situations in Class III patients and as a bone augmenting procedure in Class II patients with decreased Mandibula inclination (30, 31). Genioplasty can be performed either simultaneous to bimaxillary surgery or during the clinical course after 6 months (32). In the latter case, it is again useful to perform a further Brons-Mulié analysis to assess the chin.

## 5 Conclusions

Brons-Mulié analysis provides a simple tool for esthetic assessment of the facial soft tissue profile in addition to standard cephalometric analysis, which can be useful prior to orthognathic surgery. The postoperative application of the Brons-Mulié analysis can help to assess the quality of outcome and to identify possible planning deficits in orthognathic surgery. Often, a chin correction is an excellent additional procedure to fulfil remaining facial treatment objective after correction of the dentoskeletal mal-relation.

## Data Availability

The raw data supporting the conclusions of this article will be made available by the authors, without undue reservation.
