# Supplementary material for: The Brons-Mulié analysis as a decision-making tool for preoperative surgical simulation in orthognatic surgery
Source: Front Oral Health. 2025 Feb 19;6:1511342. doi: 10.3389/froh.2025.1511342 (PMC11887509; doi:10.3389/froh.2025.1511342)
Supplement: Supplementary file 1 [file Datasheet1.pdf]

The Brons-Mulié analysis as a decision-making tool for preoperative surgical simulation in Orthognatic Surgery

Supplement

Performing a Brons-Mulié analysis on a profile photograph

The original Brons-Mulié analysis with the underlying esthetic considerations according to facial harmony and resulting calculations has been published by Brons and Mulié (8). A mathematically simplified and clinically applicable form derived from this, which was also used in this study, enables clinical application without requiring sophisticated calculations.

To perform the Brons-Mulié analysis on a profile photograph, first of all the nasofrontal line is drawn as a tangent from the tip of the nose across the forehead. The individual vertical analysis line (VAL) is drawn caudally through the point Nasion (N) at an angle of 15 degrees. For the vertical analysis, the perpendicular projections of the points Subnasale (Sn), Stomion (St) and Menton (Me) are marked on this line. The distance between the points N and Sn on VAL determines the nasofacial height (NasFH), the distance between the points Sn and St the maxillofacial height (MaxFH) and the distance between St and Me the mandibulofacial height (MandFH). The ratio of the MaxFH and the NasFH is used to calculate the nasomaxillary ratio (VH). Using this value, an optimal value (NF / Normalface) for the mandibulofacial height (MandFH) can be calculated from the nasofacial height using the factors of Supplement table 1. In the same way, a lower (SF / Shortface) and an upper (LF / Longface) threshold can be calculated by multiplying the NasFH with the corresponding factors (Supplement Table 1). The distances NF, SF and LF are plotted on VAL and compared with the actual mandibulofacial height which enables a classification of the lower face in the vertical dimension.

To assess the sagittal dimension, the nasal bridge inclination (NRI) is drawn as the line connecting the tip of the nose with the nasion point and then the angle between NRI and VAL is determined. This value must be taken into account for the further assessment of the inclination of the lips and mandible. To assess the lips, Sn has to be connected to the most anterior point of the upper lip (OLI) and the lower lip (ULI). The angles of these two lines with VAL indicate the inclination of the upper and the lower lip, for which Brons-Mulié Analysis specifies an upper and a lower threshold which both depend on NRI (Supplement Table 2). Finally, the line connecting the pogonion to the most anterior point of the lower lip (MI) has to be drawn to assess the inclination of the mandible. The angle of MI with VAL has to be determined and compared with an upper and lower threshold which also depends on NRI (Supplement Table 2).

Supplement Table 1: Vertical Brons-Mulié analysis. The nasomaxillary ratio (VH) is calculated by dividing the maxillofacial height (MaxFH) by the nasofacial height (NasFH). The optimum value for the mandibulofacial height as well as the upper and lower thresholds are obtained by multiplying NasFH by the factors NF (normal face), SF (short face) and LF (long face) with respect to the calculated VH.

**Short Nose / Long upper lip**				**Normal Nose / normal lip**				**Long Nose / Short upper lip**			
**VH**	SF	NF	LF	**VH**	SF	NF	LF	**VH**	SF	NF	LF
**0.67**	1.06	1.04	1.02	**0.50**	0.87	0.92	0.96	**0.39**	0.74	0.83	0.93
**0.66**	1.05	1.03	1.01	**0.49**	0.86	0.91	0.96	**0.38**	0.73	0.83	0.93
**0.65**	1.04	1.02	1.01	**0.48**	0.84	0.90	0.96	**0.37**	0.71	0.82	0.93
**0.64**	1.03	1.02	1.01	**0.47**	0.83	0.89	0.95	**0.36**	0.70	0.81	0.92
**0.63**	1.01	1.01	1.00	**0.45**	0.81	0.88	0.95	**0.35**	0.69	0.80	0.92
**0.62**	1.00	1.00	1.00	**0.44**	0.80	0.87	0.94	**0.34**	0.68	0.80	0.92
**0.61**	0.98	1.00	0.99	**0.43**	0.79	0.86	0.94	**0.33**	0.67	0.79	0.91
**0.60**	0.98	0.99	0.99	**0.42**	0.78	0.86	0.94	**0.32**	0.66	0.78	0.91
**0.59**	0.97	0.98	0.99	**0.41**	0.76	0.85	0.94	**0.31**	0.64	0.77	0.90
**0.58**	0.95	0.97	0.99	**0.40**	0.75	0.84	0.93	**0.30**	0.63	0.76	0.90
**0.57**	0.94	0.97	0.98					**0.29**	0.61	0.76	0.89
**0.56**	0.93	0.96	0.98					**0.28**	0.60	0.75	0.89
**0.55**	0.92	0.95	0.98					**0.27**	0.59	0.74	0.89
**0.54**	0.91	0.94	0.97					**0.26**	0.58	0.73	0.88
**0.53**	0.90	0.94	0.97					**0.25**	0.57	0.72	0.88
**0.52**	0.89	0.93	0.97								
**0.51**	0.88	0.92	0.97								
**0.67**	1.06	1.04	1.02								

Supplement Table 2: Sagittal Brons-Mulié analysis. The inclination of the lips and mandible are determined as angles to the vertical analysis line (VAL). Depending on the nasal bridge inclination (NRI), Brons and Mulié specify threshold values for the inclinations: Lower and upper threshold of the Upper Lip Inclination (OLI: L and OLI: H), Lower Lip Inclination (ULI: L and ULI: H) and Mandibula Inclination (MI: L and MI: H).

**NRI**	**OLI: L**	**OLI: H**	**ULI: L**	**ULI: H**	**MI: L**	**MI: H**
**22.5**	-15	0	-22.5	0	-30	0
**23**	-14.5	0.5	-22	0.25	-29.5	0.25
**24**	-13.5	1.5	-21	0.75	-28.5	0.75
**25**	-12.5	2.5	-20	1.25	-27.5	1.25
**26**	-11.5	3.5	-19	1.75	-26.5	1.75
**27**	-10.5	4.5	-18	2.25	-25.5	2.25
**28**	-9.5	5.5	-17	2.75	-24	2.75
**29**	-8.5	6.5	-16	3.25	-23.5	3.25
**30**	-7.5	7.5	-15	3.75	-22.5	3.75
**31**	-6.5	8.5	-14	4.25	-21.5	4.25
**32**	-5.5	9.5	-13	4.75	-20.5	4.75
**33**	-4.5	10.5	-12	5.25	-19.5	5.25
**34**	-3.5	11.5	-11.5	5.75	-18.5	5.75
**35**	-2.5	12.5	-10	6.25	-17.5	6.25
**36**	-1.5	13.5	-9	6.75	-16.5	6.75
**37.5**	0	15	-7.5	7.5	-15	7.5

Power analysis for achievement of facial harmony

The studied cases were not based on a randomized sample, but rather on all patients available during the study period. Thus, the number of patients in this study was predetermined with n=160. The preoperative frequency of the presence of facial harmony (p1) was also predetermined. Power analysis was performed on the basis of Fisher's exact test with an alpha of 5% and an odds ratio of 2, which can be regarded as clinically relevant. In this way, a power of 0.62 to 0.86 could be achieved by varying p1 (Supplement Table 3).

Supplement table 3: Power analysis for achievement of facial harmony: In the analysis of 160 patients, with an alpha of 5%, and an odds ratio of 2, which results in a difference of 0.12 to 0.17, dependent on the level of preoperative facial harmony (p1), a power of 0.62 to 0.86 can be achieved. P2: postoperative frequency of facial harmony.

p1 (pre)	p2(post)	Difference	Power
0.4	0.57	0.17	0.84
0.5	0.67	0.17	0.86
0.6	0.75	0.15	0.8
0.7	0.82	0.12	0.62
